# Fast venous return in inferior mesenteric vein: a new sign for rectosigmoid carcinoma on CT

**DOI:** 10.1186/1470-7330-14-S1-P22

**Published:** 2014-10-09

**Authors:** A Mahfouz, H Sherif, A Sayedin, M El-Matbouly

**Affiliations:** 1 Hamad Medical Corporation, Doha, Qatar

## Purpose

Diagnosis of colorectal carcinoma on contrast-enhanced CT relies on demonstration of rectosigmoid wall thickening, perirectal fat stranding, and perirectal lymph nodes. Wall thickening may be the only sign in early carcinoma, and may be mimicked by spasm or adherent faecal matter. Rectosigmoid carcinoma may result in earlier venous return in inferior mesenteric vein (IMV) compared to superior mesenteric vein (SMV). This study evaluates faster venous return of intravenous contrast agent in IMV as a diagnostic sign for colorectal carcinoma.

## Material and methods

CT of 35 patients with colorectal carcinoma and 35 control patients have been reviewed in consensus by two experienced blinded radiologists, who reviewed CT for rectosigmoid wall thickening, lymph nodes and earlier venous return in IMV (positive IMV sign). IMV diameter and the IMV/SMV enhancement ratio have been measured and compared in the two groups by the Student’s T-test.

## Results

Sensitivity, specificity, positive predictive value, negative predictive value, and accuracy of the IMV sign for diagnosis of carcinoma have been 83, 100, 100, 89, and 93% respectively as compared to 100, 84, 81, 100, and 91% for rectal wall thickening and 40, 98, 93, 70, and 74 % for nodal enlargement respectively. IMV/SMV enhancement ratio on the arterial phase has been significantly higher in the carcinoma group (1.38±0.42) compared to the control group (0.68±0.25) (p<0.05).

## Conclusion

The IMV sign is a useful sign for the diagnosis of colorectal carcinoma, particularly in early tumours manifested only by subtle rectosigmoid wall thickening.

